# Ocular mycobacteriosis—dual infection of *M. tuberculosis* complex with *M. fortuitum* and *M. bovis*

**DOI:** 10.1186/s12348-016-0121-0

**Published:** 2017-01-13

**Authors:** Kusum Sharma, Natasha Gautam, Megha Sharma, Mohit Dogra, Priya Bajgai, Basavaraj Tigari, Aman Sharma, Vishali Gupta, Surya Prakash Sharma, Ramandeep Singh

**Affiliations:** 1 Department of Microbiology, Post Graduate Institute of Medical Education and Research, Sector 12, Chandigarh, India; 2 Advanced Eye Centre, Post Graduate Institute of Medical Education and Research, Sector 12, Chandigarh, 160012 India; 3 Department of Internal Medicine, Post Graduate Institute of Medical Education and Research, Sector 12, Chandigarh, India

**Keywords:** *Mycobacterium tuberculosis*, Nontubercular mycobacteria, PCR, Subretinal granuloma, Dual infection, *Mycobacterium tuberculosis* complex, Antitubercular therapy

## Abstract

**Background:**

We report unfavorable outcome in a patient with subretinal granuloma caused by dual infection of *Mycobacterium tuberculosis* complex with *Mycobacterium fortuitum* and *Mycobacterium bovis* in an immunosuppressed, non-HIV patient. We did a systematic review of literature on dual infection due to *M. tuberculosis* and *M. fortuitum* via MEDLINE and PUBMED and could not find any case reported of causing this kind of dual infection in the eye.

**Results:**

A 38-year-old Indian male patient presented with decreased vision in the left eye for 3 months, diagnosed as tubercular choroidal granuloma with associated retinal angiomatosis proliferans (RAP) lesion. He also had multiple enlarged lymph nodes in the chest, and sternal pus sample was positive for acid-fast bacilli (AFB). *M. tuberculosis* complex was detected by gene expert. The patient was started on antitubercular treatment (ATT) whereby the lung lesions improved but the ocular lesion showed initial clinical improvement followed by worsening. Twenty-five-gauge diagnostic pars plana core vitreous surgery was done whereby sample demonstrated a large number of AFB on Ziehl-Neelsen stain and auramine-rhodamine stain. The vitreous sample showed growth on routinely inoculated mycobacteria growth indicator tube (MGIT) 960 tubes, and multiplex polymerase chain reaction (PCR), Gene Xpert MTB/ RIF assay (Cepheid, Sunnyvale, CA), and line probe assay (LPA) were positive for ocular tuberculosis. In view of nonresponse to conventional ATT, a suspicion of dual infection of *M. tuberculosis* complex with a nontubercular mycobacteria was kept and a subculture was made onto the solid Lowenstein-Jensen (LJ) medium from the positive MGIT 960 tubes. Two morphologically distinct types of colonies were obtained on LJ slopes. Subsequently, the two etiological agents were identified as *M. fortuitum* and *M. bovis* by PCR from the vitreous sample.

**Conclusions:**

Co-infection of *M. tuberculosis* complex with nontubercular mycobacterium (NTM) has never been reported from ocular tuberculosis before. In immunosuppressed individuals, who test positive for MTB, not responding to the standard ATT, one needs to have a high index of clinical suspicion to rule out associated NTM infection and initiate appropriate multidrug systemic antibiotic therapy early.

## Findings

### Introduction

Nontubercular mycobacterium (NTM) infections are difficult to identify thus causing a delay in the diagnosis and treatment [1]. The agents causing NTM infections are found ubiquitously in soil, dust, and water. Human infection is thought to be acquired from environmental exposures [2]. In the literature, only nine cases of uveitis caused by NTM have been reported. Of these, five were detected in immunocompromised patients [1]. We report a novel case of subretinal granuloma caused by dual infection with *M. tuberculosis* complex and *M. fortuitum* leading to an unfavorable outcome in a non-HIV patient.

### Case report

**Fig. 1:**
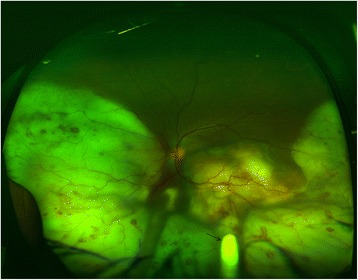
Fundus photograph of the left eye showing a large elevated mass lesion, sparing the superior part of retina, with massive exudation around the lesion and with an Ozurdex implant in situ (*black arrow*)

A 38-year-old Indian male presented with decreased vision in the left eye for 3 months. His vision was 6/6 in the right eye and 3/60 in the left eye. The intraocular pressure with Goldmann applanation tonometery was 16 and 18 mmHg in the right and left eye, respectively. Examination of the right eye was normal. Examination of the left eye revealed clear cornea with 2+ cellular reaction, 1+ flare, 1+ vitritis, and a large elevated mass lesion with massive exudation (Fig. 1). Fundus flourescein angiography revealed early blocked fluorescence in the lesion, network of retinal vessels forming retinal angiomatosis proliferans (RAP) lesions with late pooling of dye. We kept a strong possibility of tubercular choroidal granuloma with associated RAP lesion based on the above clinical findings.

Patient was a farmer by occupation. Past history revealed treatment with pulse dose of intravenous steroids followed by oral steroids at 1 mg/kg body weight, dexamethasone intravitreal implant (Ozurdex, Allergan), and co-trimoxazole for presumed ocular toxoplasmosis. This long-term use of systemic steroids at a high dosage leads to an immunosuppressed state thereby leading to a disseminated form of infection. Past investigations like complete hemogram, VDRL, HIV, HBsAg, Toxo IgG /IgM, and Mantoux test were normal. At our center, examination by internist revealed normal examination except for erythematous area with small pustule on the sternal aspect of chest. Contrast-enhanced computed tomography (CECT) of chest showed loculated pericardial effusion in anterior part along with small foci of subsegmental collapse and bronchiectasis in the left upper lobe, paracardiac part of the right medial lobe and left lingual along with few subcentimeter lymph nodes in the right paratracheal chain. Sternal pus sample was drawn which showed acid-fast bacilli (AFB) on Ziehl-Neelsen staining, and *Mycobacterium tuberculosis* complex (MTBC) was detected by gene expert.

**Fig. 2:**
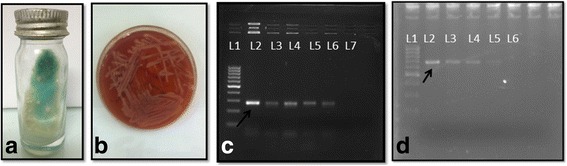
**a** Formation of moist colonies on LJ medium seen within 3 days of incubation. **b** Tiny pink colonies formed by the rapid growers on Mac Conkey agar. **c** Presence of *M. fortuitum* confirmed by using PCR targeting specific primers for *M. fortuitum. L1*—100 bp molecular marker, *L2*—275 bp positive control of *M. fortuitum (black arrow)*, *L3* and *L4*—DNA extracted from culture and positive for *M. fortiuitum*, L5 and L6—DNA extracted from patient VF sample positive for *M. fortuitum*, *L7*—negative control. Presence of *M. fortuitum* confirmed by using PCR targeting specific primers for *M. fortuitum* (*black arrow*). *L1*—100 bp MM, *L2*—positive control (*black arrow*), *L3* and *L4*—DNA extracted from culture isolate from VF sample of patient, *L5*—DNA extracted from VF sample of patient, *L6*—negative control. **d** Presence of *M. bovis* confirmed by using PCR targeting the specific *Hup B* genes. *L1*—100 bp MM, *L2*—positive control for *M. bovis* (*black arrow*), *L3* and *L4*—DNA extracted from culture of *M. tuberculosis* complex, *L5*—DNA extracted from VF of patient sample, *L6*—negative control

The patient was started on antitubercular treatment (ATT), i.e., rifampicin 600 mg, isoniazid 300 mg, pyrazinamide 1500 mg, ethambutol 800 mg, and pyridoxine 10 mg. In view of presence of dexamethasone implant (Ozurdex, Allergan), oral steroids were not given. Intravitreal bevacizumab was given for RAP lesion. In the next 2 months, there was a gradual resolution of exudative detachment with BCVA improvement. Complete resolution of the sternal lesion was noticed. Two weeks later, he presented with drop in vision and re-appearance of exudation. Clinical impression of paradoxical worsening was made. He was put on oral steroids along with ATT owing to worsening in next 3 days despite treatment. Two weeks later, 25-gauge diagnostic vitreous surgery was done. Large number of AFB on Ziehl-Neelsen stain and auramine-rhodamine stain were noticed. The conventional mycobacteria growth indicator tube (MGIT) 960 system also showed positive growth. The presence of MTBC was confirmed by multiplex polymerase chain reaction (PCR) [3], Gene Xpert, and line probe assay (LPA). It was reported as sensitive to rifampicin by Gene Xpert and sensitive to both rifampicin and isoniazid by LPA. On subculture from MGIT bottle onto Lowenstein-Jensen (LJ) medium, it grew nontubercular mycobacteria (Fig. 2a) confirmed by acid-fast staining. Subsequently, standard biochemical tests were carried out along with further subculture on 2 LJ slants and Mac Conkey medium. The organism was found to be a rapid grower and formed tiny pink colonies on Mac Conkey (Fig. 2b). Presumptively, the organism was identified as *M. fortuitum* using standard biochemical reactions like nitrate reduction and 68 °C catalase. For further confirmation, the isolate was subjected for PCR targeting *M. fortuitum* complex specific SOD gene primers and yielded a PCR product of 275 bp as described previously [4] (Fig. 2c). For PCR, 5 ml eluted DNA from culture of *M. fortuitum* and patient’s vitreous fluid (VF) sample was used. For PCR amplification with 1× PCR buffer (10 mM Tris with 15 mM MgCl2), 200 mM deoxynucleotide, 1 mM of each primer (forward primer 5′CCAAGCTCGATGAGGCGCGG 3′ and reverse primer 5′CCGATCGCCCAGGTCTGT3′), and 1 unit of Taq polymerase (Bangalore Genei, Bangalore, India). Following cycling conditions were used denaturation at 95 °C for 5 min, 35 cycles consisting of denaturation at 94 °C for 15 s, annealing at 60 °C for 15 s, and extension at 72 °C for 10 min. DNA extracted from ATCC *M. fortuitum* was used as positive control, and PCR grade water was used as negative control. Positive results yielded 275 bp PCR product in positive control, DNA extracted from *M. fortuitum* culture and DNA extracted from patient’s VF as shown in Fig. 2c. PCR product of *M. fortuitum* was confirmed by sequencing. Interestingly, the subculture made onto the solid LJ medium (Fig. 2a) from the MGIT positive tubes showed presence of relatively moist colonies within 3 days of incubation. These colonies of a “rapid-grower” on LJ slopes, along with the fact that the patient was not responding to conventional ATT, were strong pointers towards a dual infection. The rapid grower formed tiny pink colonies on Mac Conkey agar (Fig. 2b) and were presumptively identified as *M. fortuitum* on the basis of nitrate reduction and 68 °C catalase. They were later confirmed using PCR targeting specific primers for *M. fortuitum* [4] (Fig. 2c). When the LJ sloped were allowed to incubate for extended period, rough buff-colored colonies also appeared along with *M. fortuitum* colonies after 14 days of incubation. As this second etiological agent was already known to belong to MTBC (by Gene Xpert and LPA), specific PCRs for common agents of MTBC were carried out. It was confirmed as *M. bovis* using specific *Hup B* gene, as described previously [5] (Fig. 2d). On 24-locus MIRU-VNTR typing [6], using reference database and analysis available at www.miru-vntrplus.org, the MTBC isolate was found to be of the CAS-Delhi type.

Oral levofloxacin 500 mg twice a day was added, since the rapid growers like *M. fortuitum* is found to be sensitive to flouroquinolones (third generation) in addition to the ATT [2]. PET scan revealed intense FDG uptake in the affected eyeball and left supraclavicular, mediastinal, right upper, and lower paratracheal and bilateral hilar lymph nodes. In view of the poor response to treatment, he was investigated to rule out immunosuppression. His CD4 count was very low (2.8%, absolute count—51/μl), with absolute lymphocyte count of 1807/μl. Repeat HIV and HIV-1 proviral DNA were negative. Steroids were stopped, and ATT was continued for a total duration of 1 year. However, we were not able to save the eye anatomically and functionally. At 9 months of follow-up, the left eye had no light perception with phthisis bulbi and the other eye was normal. Repeat PET scan revealed no FDG uptake in the previously involved tissues. Oral isoniazid and rifampicin were continued, while oral levofloxacin was stopped.

### Discussion

In a highly TB-endemic country like ours, ocular tuberculosis is relatively not a rare entity and it occurs mainly in the form of posterior uveitis. Early detection, diagnosis, and appropriate conservative management with antitubercular therapy often yield good results in terms of preservation of vision and restoration of anatomical integrity [7]. Our patient had a large tubercular choroidal granuloma, which tested positive on Ziehl-Neelsen staining and PCR, but with a poor clinical outcome despite initiating ATT. An important reason for the poor outcome could be infection due to the multidrug resistant *M. tuberculosis*, which was ruled out, by the LPA and Gene Xpert in our case. Gupta et al. [7] hypothesized that puncturing the granuloma/abscess leads to dissemination of the organisms into the vitreous cavity followed by the development of endophthalmitis that may not be amenable to treatment and may eventually require evisceration. Biswas et al. [8] reported relatively worse outcome in the eyes subjected to pars plana vitrectomy and drainage of the abscess. However, no attempt was made to either drain or puncture the granuloma during pars plana vitrectomy (PPV) in our case. Thus, we re-evaluated our case to find out the possible causes, and the samples that we obtained during PPV were again subjected to cultures and molecular diagnostics. Our patient now tested positive for a NTM along with *M. tuberculosis* complex on LJ medium, which was confirmed by PCR. Thus, the presence of dual infection and delay in its diagnosis could be the cause for poor outcome in this case. This could have further been worsened by the concomitant use of intravenous steroids, oral steroids, and dexamethasone implant resulting in low CD4 counts and a disseminated form of infection.

NTM infections are rare, mainly seen in the immunocompromised individuals and often leading to dissemination in the absence of appropriate treatment. In the literature, NTM has been shown as a cause for choroiditis in six eyes, iridocyclitis in one eye, and granulomatous panuveitis in two eyes and treatment with steroids was implicated as a risk factor in 42.9% of the cases [1]. There are four groups of NTM, among which, group IV, i.e., the rapid growers (including *M. fortuitum*), is the one most commonly infecting the eye as was seen in our case. They do not respond to conventional ATT alone and require a prolonged therapy with multidrug parenteral antibiotics [2].

Our case also highlights the diagnostic role of molecular methods for these infections in routine laboratories for rapid management and better patient outcome. The role of solid culture in identifying presence of dual infection, which would have been otherwise missed on liquid culture alone, is also important in this case, as previously reported from our center [9].

In conclusion, the diagnosis of NTM infection and the possibility of it occurring along with MTBC infection require a high index of suspicion on the part of the ophthalmologist and a rapid and reliable diagnosis on the part of the microbiologist. The use of immunosuppressant drugs, atypical features, and poor response to ATT are important clinical pointers towards this rare condition. Confirmatory evidences can be obtained from growth on solid culture media followed by appropriate molecular tests. A delay in diagnosis could lead to irreversible damage as was seen in our case, and thus, prompt initiation of multidrug therapy is often needed. Although our patient lost his vision of one eye, the relatively rapid diagnosis and timely institution of oral levofloxacin (third-generation flouroquinolones), along with stoppage of oral steroids, helped in preventing the dissemination of the infection in the body as was shown by the absence of uptake of FDG in various parts of the body on PET scan.

## Abbreviations

AFB: Acid-fast bacilli
ATT: Antitubercular treatment
CECT: Contrast-enhanced computerized tomography
FDG: Flourodeoxyglucose
LJ medium: Lowenstein-Jensen medium
LPA: Line probe assay
MTBC: *Mycobacterium tuberculosis* complex
NTM: Nontubercular mycobacterium
PCR: Polymerase chain reaction
PET scan: Positron emission tomography scan
RAP: Retinal angiomatosis proliferans
